# Diabetes and liaison psychiatry: the characteristics of patients with diabetes referred to a liaison psychiatry service in London

**DOI:** 10.1192/bjo.2021.309

**Published:** 2021-06-18

**Authors:** Alexandra Simpson, Lucy Bradford, Marilia Calcia

**Affiliations:** 1 East Sussex Healthcare NHS Trust; 2 King's College London

## Abstract

**Aims:**

To determine the characteristics of adult patients referred to a Liaison Psychiatry service in a general teaching hospital in London, UK with 950 inpatient adult beds.

**Method:**

All referrals for adult inpatient psychiatric consultation made during a period of 9 months were reviewed; those that involved a patient with a diagnosis of diabetes were analysed. Descriptive statistics were used; data were collected on demographic characteristics and physical and mental health parameters, including type of diabetes, number of years since diabetes diagnosis, glycaemic control, presence of diabetes-related complications, reason for Psychiatry consultation request, psychiatric diagnosis, psychotropic medication, frequency of admissions to general hospital, psychiatric risk issues and outcome of psychiatric consultation.

**Result:**

Pilot results indicate that 30 diabetic patients were referred for a psychiatric consultation in 9 months. Of those, 9 had type 1 diabetes, 17 had type 2 diabetes and 1had pre-diabetes 3 were unknown. 13 were male and 17 were female; the median age was 46 (range 18 to 68); the ethnicities were 6 White, 15 Black, 1 Asian and 8 other.

Diabetes-related complications were present in 77% (retinopathy 10%, kidney disease 27%, neuropathy 13%, diabetic foot 16%). 6% had comorbid cardiovascular disease. 10% were on dialysis and 3% had had amputations.

The main reason for referral for psychiatric consultation was low mood and self harm; other reasons were recurrent DKA, anxiety and self neglect. Psychiatric risk issues included 20% risk of self-harm/suicide; 13% risk of violence; 10 risk of self-neglect. The outcomes of liaison psychiatry consultation were: 30% received an assessment that led to recommendations to the general medical team and did not require further psychiatric input; 27% received continued psychiatric follow-up during the admission. With regards to treatment, 36% had psychiatric treatment (including medication) reviewed; 47% received general treatment recommendations, including recommendations for new laboratory or radiological investigations or change in level of nursing care. 20% required transfer to an inpatient psychiatric unit, with 33% discharged to care of community mental health.

**Conclusion:**

Our findings indicate the scope of practice for a Liaison Psychiatry service with regards to adult hospital inpatients with diabetes. Our data suggest that patients with type 2 diabetes are the majority of inpatients with diabetes that require psychiatric consultations, and that the majority of those are patients already known to psychiatric services due to long-term severe mental disorders, particularly schizophrenia, schizoaffective disorder or bipolar disorder. Most of those patients have medical comorbidities and severe diabetes-related complications. Patients with type 1 diabetes, despite making up a smaller proportion of referrals for psychiatric consultations, also tend to have recurrent hospital admissions and features of self-neglect.

